# ResNet1D-Based Personal Identification with Multi-Session Surface Electromyography for Electronic Health Record Integration

**DOI:** 10.3390/s24103140

**Published:** 2024-05-15

**Authors:** Raghavendra Ganiga, Muralikrishna S. N., Wooyeol Choi, Sungbum Pan

**Affiliations:** 1 Department of Information and Communication Technology, Manipal Institute of Technology, Manipal Academy of Higher Education (MAHE), Manipal 576104, India; raghavendra.n@manipal.edu; 2 Department of Computer Science and Engineering, Manipal Institute of Technology, Manipal Academy of Higher Education (MAHE), Manipal 576104, India; 3 Department of Computer Engineering, Chosun University, Gwangju 61452, Republic of Korea; wyc@chosun.ac.kr; 4 IT Research Institute, Chosun University, 309 Pilmun-daero, Gwangju 61452, Republic of Korea

**Keywords:** sEMG, ResNet1D, EHR, CNN-LSTM, healthcare, security

## Abstract

Personal identification is an important aspect of managing electronic health records (EHRs), ensuring secure access to patient information, and maintaining patient privacy. Traditionally, biometric, signature, username/password, photo identity, etc., are employed for user authentication. However, these methods can be prone to security breaches, identity theft, and user inconvenience. The security of personal information is of paramount importance, particularly in the context of EHR. To address this, our study leverages ResNet1D, a deep learning architecture, to analyze surface electromyography (sEMG) signals for robust identification purposes. The proposed ResNet1D-based personal identification approach using the sEMG signal can offer an alternative and potentially more secure method for personal identification in EHR systems. We collected a multi-session sEMG signal database from individuals, focusing on hand gestures. The ResNet1D model was trained using this database to learn discriminative features for both gesture and personal identification tasks. For personal identification, the model validated an individual’s identity by comparing captured features with their own stored templates in the healthcare EHR system, allowing secure access to sensitive medical information. Data were obtained in two channels when each of the 200 subjects performed 12 motions. There were three sessions, and each motion was repeated 10 times with time intervals of a day or longer between each session. Experiments were conducted on a dataset of 20 randomly sampled subjects out of 200 subjects in the database, achieving exceptional identification accuracy. The experiment was conducted separately for 5, 10, 15, and 20 subjects using the ResNet1D model of a deep neural network, achieving accuracy rates of 97%, 96%, 87%, and 82%, respectively. The proposed model can be integrated with healthcare EHR systems to enable secure and reliable personal identification and the safeguarding of patient information.

## 1. Introduction

**Figure 1:**
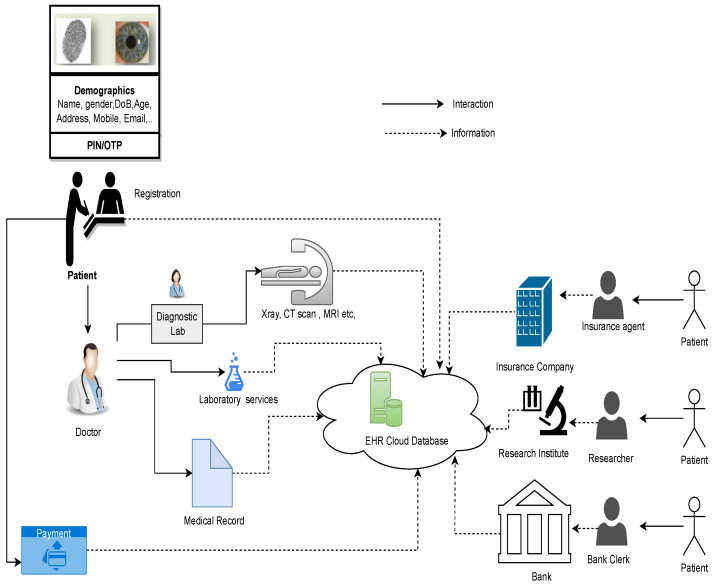
A visual overview of electronic health record (EHR) data utilization.

An electronic health record (EHR) is a digital version of a patient record, encompassing a spectrum of health information stored within the hospital’s server [1,2]. With the rapid evolution of information technology, traditional healthcare is transitioning towards a more electronic sphere. This shift is underscored by the twenty-first century’s digitization revolution, where patient records are now securely stored in cloud-based storage [3,4,5]. These digital archives have been extensively utilized by clinicians, healthcare providers, patients, and insurance companies, with the collective aim of creating, managing, and seamlessly accessing patients’ health data worldwide [6]. Additionally, these data repositories offer the potential for shared use among diverse healthcare stakeholders, enabling the monitoring of patient well-being, administration of effective treatments, and reduction in expenses. The cloud-integrated EHR system, as illustrated in Figure 1, plays a pivotal role in facilitating the effortless sharing of patient health data across various healthcare providers [7].

The data in the EHR system, comprising significant and vital information, are a patient’s asset. Preserving data secrecy is imperative, especially given the transmission of sensitive data through public channels. In the United States (U.S.), all participants within the healthcare ecosystem are mandated to uphold the security and safety regulations of the Health Insurance Portability and Accountability Act (HIPAA) during the collection, storage, and exchange of EHRs [8,9]. However, the task of maintaining system security and safeguarding privacy presents an ongoing challenge [10,11,12].

Authentication involves verifying an individual’s identity to either allow or deny access to services in a secure system. It requires recognizing the user among others in the user pool and determining whether they are legitimate members or imposters who should be blocked. This authentication is achieved through various identification techniques, such as something the user knows (e.g., passwords), something they have (e.g., identity cards), or who the user is (e.g., biometrics) [13]. Utilizing these factors ensures that only authorized users can access resources, thereby enhancing security and protecting against unauthorized access and potential breaches [14].

Biometric systems rely on recognizing users’ data by using their unique physical or behavioral features. Biometrics encompass a range of physiological and behavioral characteristics that uniquely distinguish an individual from others. In contrast to traditional user identification methods in secure systems, biometric traits offer several advantages. They eliminate issues associated with stolen or forgotten identification tools since biometric traits are inherently tied to the individual and do not require memorization [15]. Furthermore, biometric traits cannot be easily shared or replicated, providing an additional layer of security [16]. Additionally, biometrics can verify liveness, ensuring that the individual is physically present during the authentication process. These attributes make biometrics a valuable and reliable approach to user identification, addressing the limitations of traditional identification methods [17].

In biometrics, modalities can be classified into four categories based on their characteristics [18,19]. The first category is image-based, which includes biometrics such as fingerprints, iris scans, facial recognition, and hand geometry [20]. The second category is signal-based, involving biometrics like voice recognition, electrocardiography (ECG), electroencephalography (EEG), and electromyography (EMG) [21,22]. The third category is behavioral-based, determined by how individuals act or use devices, such as gait recognition, keystroke dynamics, or handwriting patterns [23]. The fourth category is pattern-based, which includes biometrics like Deoxyribonucleic Acid (DNA) analysis [24]. These modalities vary in terms of security levels, cost, and complexity. Furthermore, they exhibit different levels of immunity to fraud or reproduction attempts. Understanding these variations is essential when selecting appropriate biometric modalities for specific applications [22,25].

Biomedical signals encompass electrical signals acquired from organs, representing specific physical variables [26]. These signals are typically time-dependent and characterized by parameters such as amplitude, frequency, and phase. Among these signals, EMG measures the electrical currents resulting from muscle contractions, reflecting neuromuscular activity [27]. Since the nervous system controls muscle activity, EMG signals are intricate and influenced by anatomical and physiological muscle traits [26,28]. These signals encounter noise propagation through various tissues and, particularly at the skin’s surface, collect signals from multiple motor units, introducing potential interactions. Robust EMG signal detection with advanced methodologies holds significance in biomedical engineering [29]. The electrical currents generated during muscle contraction offer insights into neuromuscular activities and muscular morphology [30,31,32]. This paper utilizes surface EMG data for personal identification within EHR access control mechanisms, exemplifying the vital role of sEMG in enhancing patient data security and privacy. Integrating sEMG-based biometric authentication into EHR systems offers several advantages. Unlike traditional authentication methods such as passwords or tokens, sEMG-based identification is inherently tied to the individual’s physiological characteristics, making it difficult to forge or replicate. This enhances the overall security of EHR systems by reducing the risk of unauthorized access.

## 2. Related Work

This section provides an overview of the applications of sEMG based on its non-invasive recording method compared to invasive electromyography (EMG) techniques involving needle insertion into muscles [33]. In recent years, the use of sEMG has surged, surpassing invasive methods due to its non-painful and non-intrusive characteristics [34]. sEMG allows for the acquisition of muscle activation data without needle insertion, making it widely acceptable and suitable for various applications. The following sections present a comprehensive review of the diverse applications and research areas where sEMG data have been extensively utilized. These applications highlight the potential of sEMG in medical diagnostics, rehabilitation, human–computer interaction, and gesture recognition [35]. Furthermore, this review explores advancements in machine learning and signal processing techniques that play a pivotal role in extracting valuable information from sEMG signals, enabling improved analysis and interpretation.

Focusing on the application of sEMG signals for exercise fatigue detection, Sun et al. [36] explore this subject, highlighting advancements in measuring and interpreting sEMG signals as vital indicators for assessing exercise fatigue during human activities. The review emphasizes signal processing techniques, feature extraction, and classification methods specifically tailored for exercise fatigue assessment. The topics addressed include real-time detection, artifact removal, and dimensionality reduction to improve data quality and computational efficiency. The study also delves into combining processing methods and pattern recognition techniques to enhance classification speed and accuracy. Overall, this review serves as a valuable guide for future developments in sEMG-based exercise fatigue detection, providing insights into the evolving trends and potential advancements in this field.

Exploring the use of EEG signals as a human biometric for identification and authentication, Radwan et al. [37] discuss the challenges associated with EEG-based identification, which involve signal preprocessing and hand-designed features due to the complexity of EEG-signal traits and analysis. To address these challenges, the study adopts end-to-end neural networks, resulting in significant performance improvements. Three deep learning models, Convolutional Neural Network (CNN), Long Short-Term Memory (LSTM), and Gated Recurrent Unit (GRU), are applied to the BCI200 dataset, achieving impressive accuracy rates of 96.17%, 97.83%, and 96.53%, respectively. The study highlights the potential of deep learning in enhancing EEG-based person identification, particularly emphasizing the suitability of CNN, LSTM, and GRU models for this application.

Addressing the need to safeguard sensitive data stored within mobile devices against unauthorized access, Li et al. [38] explore mitigation techniques through a novel approach employing two-factor authentication. This method utilizes sEMG-based biometrics combined with pattern recognition. Through experimentation involving 10 subjects, the study demonstrates the efficacy of time-domain sEMG features and one-class classifiers, achieving commendable authentication performance indicated by robust F1 scores and a favorable Half of Total Error Rate (HTER). Beyond its application in mobile devices, the proposed methodology holds promising potential for adoption in vehicle access control systems, particularly for electric vehicles (EVs). This domain is anticipated to become a lucrative market valued at approximately USD 22.6 billion by 2027 [39]. The research contributes significantly to the critical imperative of securing mobile device data and extends its impact to the broader landscape of vehicle access control, paving the way for enhanced security measures in both spheres.

Sun et al. [40] conducted a study focusing on the development of an authentication algorithm utilizing EMG signals and artificial neural networks. The research addressed the evolving landscape of security technologies leveraging bio-signals, such as iris scans, electrocardiography, and fingerprint recognition, by proposing an EMG-based authentication algorithm to enhance existing personal certification techniques. The study integrated an artificial neural network clustering algorithm that included preprocessing, feature extraction, and classification stages. The authentication process relied on five key parameters extracted from EMG signals. An experimental validation of the proposed algorithm demonstrated its capability to distinguish identities, achieving an accuracy rate of 81.6% across subjects. This research contributed significantly to enhancing security protocols through the integration of EMG signals and advanced neural network methodologies, augmenting the strengths of existing personal certification techniques.

Shioji et al. [41] proposed a novel approach to combine hand motion recognition and personal authentication concurrently, leveraging wrist EMG signals. The methodology involved a sequential process of measurement, preprocessing, feature extraction, and identification. Hand motion recognition was segmented into three distinct classes, while personal authentication was categorized into two classes. Experimental findings demonstrated the efficacy of this approach, achieving an impressive accuracy of 94.5% for hand motion recognition and 94.6% for personal authentication. Moving forward, the authors’ future endeavors will focus on refining the model by addressing intra-individual differences and enhancing the CNN layer structure. This work laid the foundation for integrated hand motion recognition and authentication systems powered by wrist EMG signals, offering promising advancements in biometric technology.

Choudhary et al. [42] examined the utilization of electromyography (EMG) signals along with machine learning techniques for hand movement classification, with a focus on enabling a human-to-human interface to facilitate movement control for paralyzed individuals. The study involved classifying hand movements of subjects using various algorithms, including k-nearest neighbors (KNN), support vector machines, naive Bayes, and decision trees. The findings revealed that all algorithms achieved encouraging accuracy levels, averaging around 90%, except for KNN, which achieved a 60% accuracy. These results underscored the potential of machine learning algorithms to effectively control the movements of individuals with disabilities through a human-to-human interface. This research contributed significantly to the development of innovative solutions in the realm of movement control for individuals with paralysis, showcasing the promise of EMG signals and machine learning techniques in enhancing accessibility and quality of life

Yousefi et al.’s work [43] emphasizes the importance of utilizing effective EMG signals in diagnosing neuromuscular disorders. The paper provides a comprehensive overview of proficient EMG characterization and highlights state-of-the-art advancements in this domain, specifically focusing on neuromuscular pathologies. Each method’s associated findings are succinctly summarized. The review prominently showcases the pivotal roles played by artificial neural networks (ANNs) and hybrid neural networks in EMG classification, leading to highly accurate outcomes. While back-propagation generally achieves elevated accuracy, its efficacy is impeded by suboptimal learning-discrimination performance due to varying noise signals in EMG classification. Wavelet neural networks (WNNs) emerge as an ideal technique, boasting commendable accuracy and speed, and thus proving highly promising for providing clinically valuable insights. That review significantly contributes to understanding the landscape of effective EMG-based neuromuscular disorder diagnosis and highlights the potential of ANN and hybrid networks, as well as WNNs, in this context.

Previous studies on EMG signals primarily focused on hand muscles and noise reduction through preprocessing. A shift to deep learning methods integrated EMG with other biometrics to overcome limitations, often analyzing a limited range of gestures. sEMG signals are non-invasive, unlike invasive EMG signals. However, traditional sEMG databases for personal identification lack subject diversity and fail to capture signal variability. This paper introduces a method using a multi-session sEMG database to address these challenges. The literature emphasizes the value of sEMG signals across various domains, particularly highlighting neural networks like ANNs, LSTMs, and CNNs. We highlight ResNet-1D’s potential for sEMG signal analysis, especially in personal identification tasks, due to its advanced feature extraction and classification capabilities. Our research applies ResNet-1D to sEMG signal analysis to address key identification challenges, aiming to enhance accuracy and reliability in healthcare, security, and assistive technologies.

## 3. Materials and Methods

**Figure 2:**
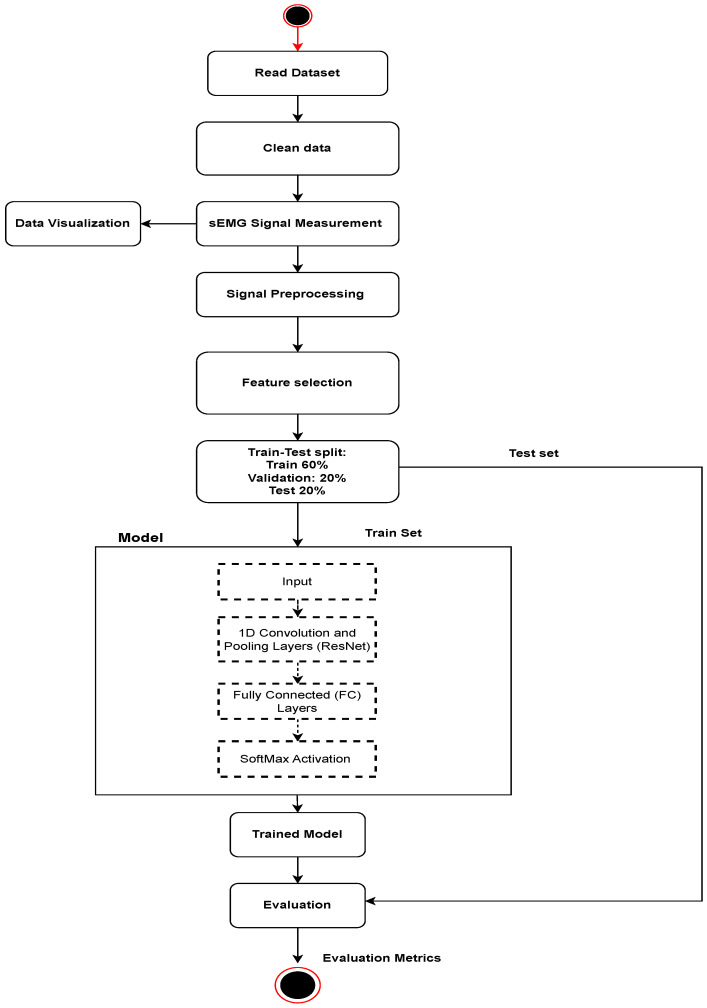
The proposed model flow diagram for experimentation.

In this research, we investigated the feasibility of using electromyogram (sEMG) readings and advanced machine learning techniques for personal identification within an electronic health record (EHR) access control mechanism. The entire process was implemented using the Python programming language, leveraging popular frameworks such as TensorFlow and Keras. Our methodology, depicted in Figure 2, consisted of key stages. Firstly, we performed data preparation by acquiring an sEMG dataset comprising 20 subjects performing 12 different gestures, categorized into static and dynamic gestures. Subsequently, we applied various data preprocessing techniques, including noise reduction, normalization, and dimensionality reduction, to optimize the sEMG data for predictive modeling. Next, we divided the dataset into training, validation, and testing sets with a ratio of 60:20:20 to ensure an unbiased evaluation of the model’s generalization on unseen data. For personal identification, we employed the ResNet1D model, a deep learning architecture well suited for sequential data like sEMG signals. The ResNet1D model was trained on the preprocessed data, and its performance was assessed using standard evaluation metrics. This research aims to contribute insights into the potential of sEMG-based identification for enhancing EHR access control mechanisms.

### 3.1. sEMG Data Description

**Figure 3:**
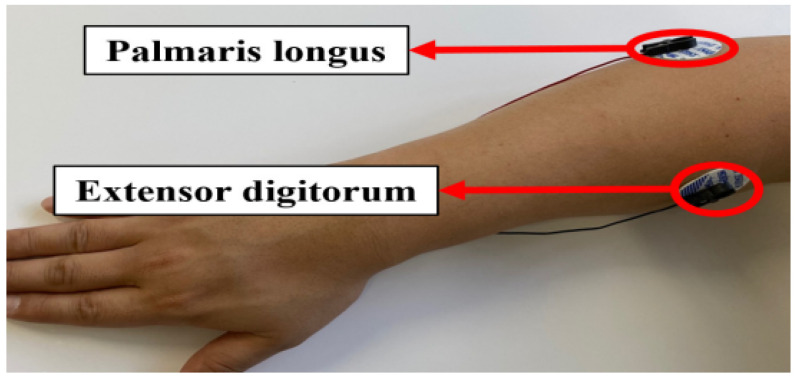
The sEMG sensor positions [44].

The constructed database (DB), sEMG, was obtained in multiple sessions from 200 subjects at intervals of a day or longer. The sEMG data were collected using the BIOPAC MP160CE system with the Acknowledge 5.0.2 processing software from the right arm of 200 subjects (98 males and 102 females), whose average age was 24.69 years (ranging from 19 to 70 years). The data were acquired in two channels by attaching Ag/AgCl sensors to the palmaris longus and extensor digitorum muscles of each subject, as illustrated in Figure 3. The sensor was placed precisely at points where significant muscle changes occur during hand gestures. A demographic survey was conducted with the participants before the signal recording process.

**Figure 4:**
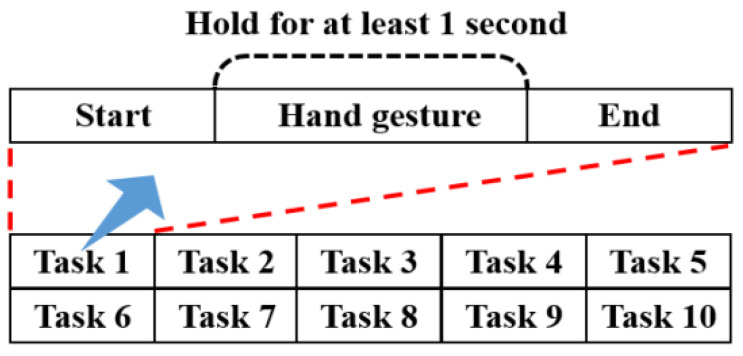
A data sequence of sEMG data acquisition for each gesture [44].

Figure 4 illustrates the process of data sequence construction for the sEMG database. Each subject performed specific movements while in a relaxed state, seated in a chair. The movements were repeated 10 times to capture multiple instances of the data. Each subject performed the hand gesture for a minimum of one second, always starting in a relaxed state and ending in a relaxed state.

**Figure 5:**
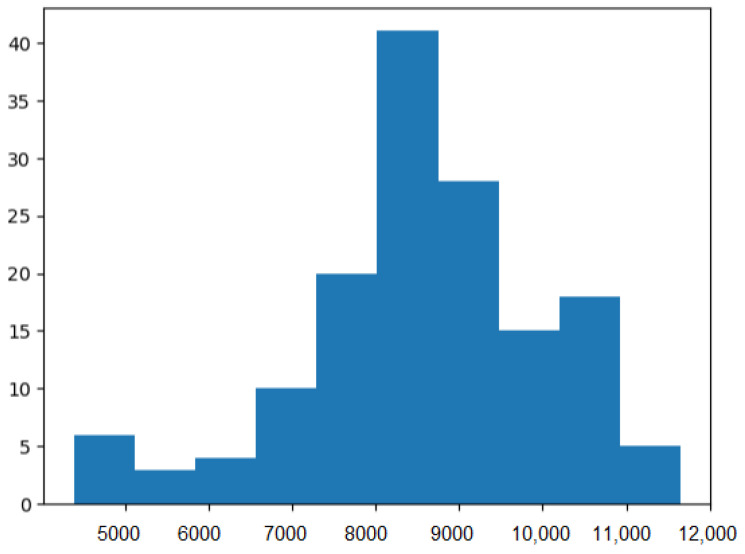
The histogram depicting signal characteristics for five selected subjects.

The recorded sEMG signals were sampled at a rate of 2000 Hz, providing a high temporal resolution. The signals were digitized with a 16-bit ADC resolution, ensuring accurate representation of the data. The subjects were selected from among students and researchers at Chosun University, and individuals not associated with Chosun. The deliberate inclusion of subjects from different backgrounds aimed to capture the variability in sEMG patterns and enhance the generalizability of the database. To provide a visual representation of the sEMG signal data, histograms depicting signal characteristics for five selected subjects are presented below in Figure 5.

### 3.2. Data Preparation

**Figure 6:**
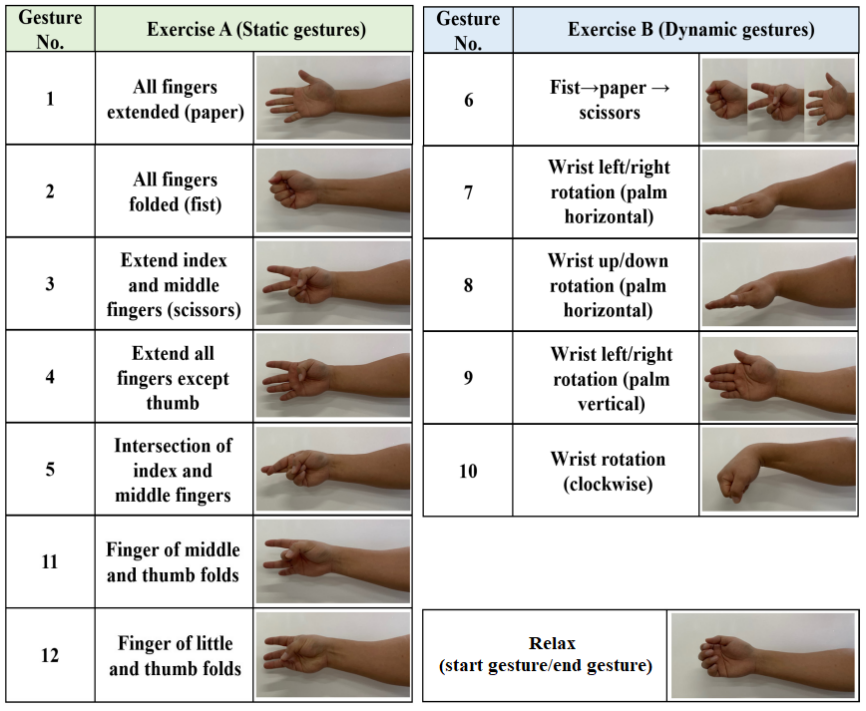
Hand gestures of CS_sEMG DB [44].

The collected sEMG dataset consisted of 20 subjects, each performing 12 different gestures, as depicted in Figure 6. These gestures encompassed both static and dynamic movements of the hand, reflecting commonly used actions in everyday life. The static gestures involved single movements, while the dynamic ones involved continuous movements. To ensure accurate and comprehensive data, the sEMG signals were recorded over three sessions, with a day or longer intervals between each session. This multi-session approach captured the variability in the sEMG signals over time. For each subject, data for each gesture were obtained in three sessions, resulting in a total of 30 sEMG signals (10 repetitions × 3 sessions) per gesture. Consequently, each subject contributed 360 sEMG signals (12 gestures × 10 repetitions × 3 sessions) in total.

When we collected sEMG datasets for our database, there were many difficulties in maintaining signal quality. In particular, variation in the magnitude of the signal intensity and the shape (envelop) of the signal within the contraction period was the critical factor for data verification. Therefore, we set up guidelines for acquiring qualified signals. For example, if there was a signal whose magnitude was 20% higher than the others, we removed the signal and extracted it again. Likewise, we replaced the signal whose envelope differed from others with the newly acquired signal. As a result, we were able to keep the EMG signal characteristics as consistent as possible when building our database. We informed each of the participants of the course of the acquiring process beforehand so that the participants appreciated such repetition of muscle actions.

**Figure 7:**
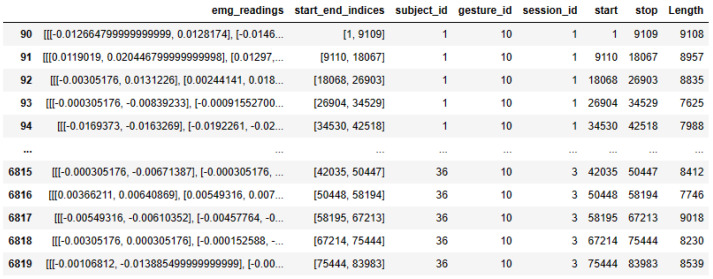
The sEMG DB files’ structure information.

Figure 7 illustrates detailed information about the sEMG data, including column information. It consists of five columns. Column 1 contains information about the 2-channel sEMG data. Column 2 provides start and end indices related to sampling information. Column 3 contains subject ID details for the performed gesture, while Column 4 indicates the gesture number performed by the subject. Finally, Column 5 contains session IDs and details for the three sessions. This comprehensive dataset provides a robust foundation for further analysis and exploration of gesture identification using sEMG signals.

**Figure 8:**
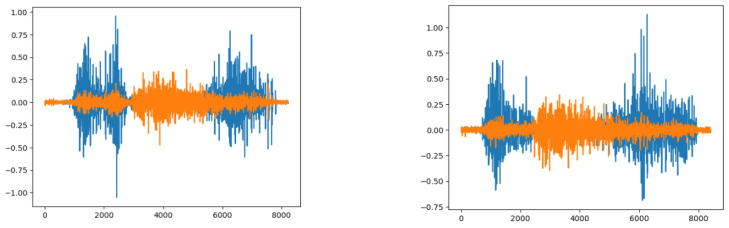
Illustration of the differences in sEMG signals for the same subject and gestures (Subject 5, Gesture 10) across two trials, emphasizing the intra-class variability. The signals are depicted using two channels, represented by the blue and orange plots.

The constructed sEMG database used for this study consisted of 200 subjects. However, 84 subjects were excluded from the analysis due to various reasons such as “Device loses Bluetooth connection”, “Incompletion of muscle contraction/relaxation”, “Insufficient number of repetitions”, and “Muscle not activated”. These exclusions were made based on the criteria mentioned in the cited paper. Figure 8 illustrates the results obtained from the sEMG signals for Gesture 10 of Subject 5, visually representing the findings. The constructed sEMG database is publicly available at the IT Research Institute of Chosun University, accessible through their website (http://www.chosun.ac.kr/riit, accessed on 21 February 2022). The unfiltered raw sEMG data are provided in text files and MATLAB files. However, only the EMG data of Gestures 1 to 3 performed by 100 subjects, including those excluded due to errors, are disclosed for non-commercial purposes. Access to the complete dataset requires signing a Memorandum of Understanding (MOU) with the IT Research Institute of Chosun University. The text files contain sEMG signals without notions of repetitions, while the MATLAB files include sEMG signals with notions of repetitions [44].

### 3.3. Signal Preprocessing

Signal processing is an essential stage of the sEMG signal analysis, aiming to remove unwanted noise and interference to obtain a cleaner and more informative signal for further processing and analysis. Here is a summary of the filtering process and its purpose:

- Baseline wander removal: Baseline wander refers to low-frequency fluctuations in the sEMG signal caused by breathing, slight movements, or other non-electrophysiological sources. By using a bandpass filter with a minimum frequency of 5 Hz, the baseline wander is attenuated, reducing its influence on the signal [45].
- Power line noise removal: Electrical devices and power lines emit electrical signals at the power line frequency, which can contaminate the sEMG signal. In many regions, the power line frequency is 60 Hz. The bandpass filter with a notch at 60 Hz effectively removes this power line noise from the signal [46].
- External environment noise removal: sEMG signals can also be affected by external noise sources, such as electromagnetic interference or muscle noise from adjacent muscle groups. The bandpass filter with its selected frequency range helps to minimize the impact of these external noise sources [47].
- Frequency range selection: the bandpass filter retains frequencies between 5 Hz and 500 Hz, which are typically relevant for capturing muscle activity signals while excluding frequencies outside this range that might not be related to the desired sEMG data.
- Filter order: A filter’s order of 3 determines the sharpness of the filter’s roll-off characteristics. A higher-order filter allows for a steeper reduction in frequencies outside the passband, providing more effective noise removal [48].

By applying this bandpass filter to the sEMG signal with a sampling frequency of 2000 Hz, the preprocessing stage effectively enhanced the quality of the data by eliminating noise and unwanted frequency components. This processed signal can then be used for various analyses, such as feature extraction, classification, or any other predictive modeling tasks.

### 3.4. Analyzing the ResNet1D Model for Personal Identification

A residual block in a neural network incorporates shortcut connections, also known as skip connections, which allow the input of a layer to be directly added to the output of a subsequent layer. This approach enables the network to focus on learning the residual (the difference between input and output) rather than the entire transformation, simplifying the learning process for deeper layers. ResNet was designed to address the issue of vanishing gradients, which can impede training in very deep networks. As the network depth increases, gradients may diminish, posing challenges in weight updates and effective model training [49].

To adapt ResNet to 1D data, such as time series or sequences, adjustments are made to accommodate the 1D input format. Each residual block comprises several components, including a BatchNorm layer for data normalization from the previous layer, a ReLU layer for introducing a non-linear activation, a dropout layer for regularization, and a convolutional layer for feature extraction. Additionally, a Maxpool1D layer is included in the first sub-blocks of every block.

**Figure 9:**
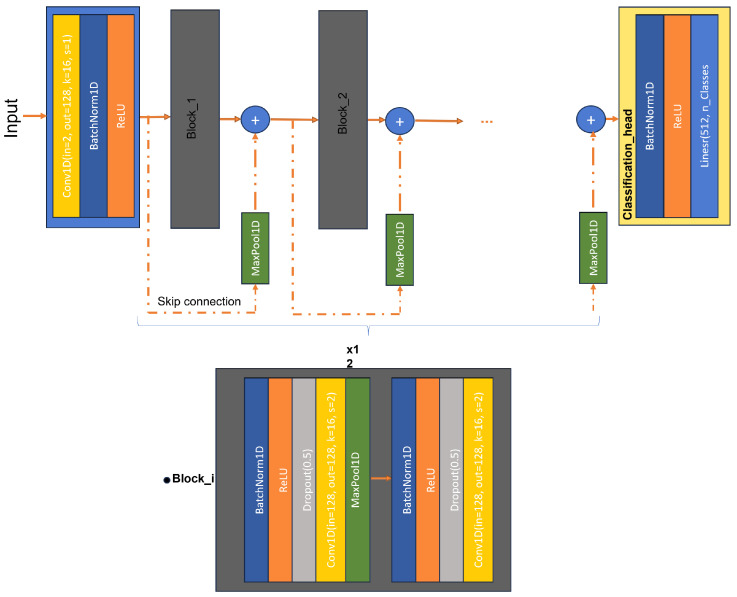
The architecture of the proposed model for the experimentation.

Figure 9 illustrates the employed residual neural network (ResNet-1D) for personal identification. The complete structure consists of 12 stacked blocks with skip connections, and a classification head is attached to predict the class label. The arrangement of layers within any Block_i in the residual network is also depicted. This combination of layers enhances the network’s performance and facilitates effective training on 1D data.

## 4. Results and Discussion

The dataset comprised sEMG recordings obtained from the right arms of 200 subjects, each performing 12 gestures. The sEMG signals were recorded using Ag/AgCI electrodes placed over the muscles of interest. The dataset was split for training and testing purposes. Although data were collected from 200 subjects, we randomly sampled 20 instances for training and testing. The dataset size was substantial, making it impractical to use all samples for training and testing.

### 4.1. Model Performance Metrics

In evaluating the performance of the ResNet1D model for personal identification, key metrics such as accuracy, precision, recall (sensitivity), and F1 score were employed. These metrics assess overall correctness, precision of positive predictions, true positive rate, and a balanced measure of precision and recall, respectively. The equations used to calculate these metrics are referenced as Equations (1)–(4). Accuracy quantifies the overall correctness of our model’s predictions, calculated as the ratio of the number of correct predictions (true positives and true negatives) to the total number of predictions made. Precision assesses the accuracy of positive predictions by computing the ratio of true positives (TP) to the sum of true positives and false positives (FP), focusing on minimizing false positive identifications. Recall (or sensitivity) gauges the model’s ability to detect all positive instances by calculating the ratio of true positives (TP) to the sum of true positives and false negatives (FN), ensuring a comprehensive identification of relevant positive cases. The F1 score, a balanced measure of precision and recall, harmonizes these metrics to provide a comprehensive evaluation of our ResNet1D-based approach in personal identification, particularly valuable for seamless integration with electronic health records using multi-session sEMG data. The acceptable standards for accuracy, precision, recall, and F1 score depend on various factors such as the specific problem domain, the consequences of false positives and false negatives, and the overall goals of the task. However, in general, there are no fixed universally accepted thresholds for these metrics. Instead, they are typically determined based on the specific requirements and constraints of the problem at hand.

(1) $$\mathrm{Accuracy}=\frac{\mathrm{Number}\;\mathrm{of}\;\mathrm{correct}\;\mathrm{predictions}}{\mathrm{Total}\;\mathrm{number}\;\mathrm{of}\;\mathrm{predictions}}$$

(2) $$\mathrm{Precision}=\frac{\mathrm{TP}}{\mathrm{TP}+\mathrm{FP}}$$

(3) $$\mathrm{Recall}\;\left(\mathrm{Sensitivity}\right)=\frac{\mathrm{TP}}{\mathrm{TP}+\mathrm{FN}}$$

(4) $$F1\;\mathrm{Score}=2\times \frac{\mathrm{Precision}\times \mathrm{Recall}}{\mathrm{Precision}+\mathrm{Recall}}$$

The performance evaluation of our ResNet1D-based approach for personal identification using sEMG signals yielded promising results across four different scenarios, each conducted on a dataset of 20 subjects. Notably, the identification accuracy achieved in these experiments was exceptional. The experiments were conducted separately for 5, 10, 15, and 20 subjects using the ResNet1D model of a deep neural network, and the results are presented in Table 1 and Table 2.

For the case of five subjects, our model achieved an impressive accuracy of 92% on the test set. Similarly, for 10, 15, and 20 subjects, the accuracy reached 96%, 87%, and 82%, respectively. These accuracy scores demonstrate the effectiveness of our approach in accurately distinguishing individuals based on their unique sEMG patterns.

Furthermore, the precision scores for the different cases were consistently high, showcasing the model’s ability to precisely predict positive instances for each subject. For 5, 10, 15, and 20 subjects, the precision scores were 0.97, 0.96, 0.90, and 0.88, respectively. These scores indicate that a significant proportion of individuals predicted as positive were correctly identified, underscoring the reliability of the ResNet1D model in personal identification tasks.

Moreover, the model demonstrated a high sensitivity in identifying actual positive instances, as evidenced by the recall scores. For 5, 10, 15, and 20 subjects, the recall scores were 0.97, 0.95, 0.86, and 0.83, respectively. This means that the model successfully detected a substantial percentage of the individuals who should have been identified, further validating its robustness in personal identification.

The F1 scores, which balance precision and recall, were consistently high across the different cases, with values of 0.97, 0.96, 0.86, and 0.83 for 5, 10, 15, and 20 subjects, respectively. This indicates the model’s well-rounded performance in correctly identifying individuals while minimizing false negatives. In the context of the weighted average, the calculation involves determining the metric for each class individually. However, each class is assigned a weight proportional to its prevalence in the dataset. This weighting mechanism is applied to address potential imbalances among different classes. In our case, we had a balanced dataset. Thus, the macro average ensured an equal treatment of all classes, irrespective of their support values.

Overall, these results reinforce the effectiveness of our ResNet1D-based approach for personal identification using sEMG signals. The high accuracy, precision, and recall scores suggest that the model can reliably differentiate individuals based on their unique sEMG patterns, making it a promising solution for real-world applications in healthcare, security, and assistive technologies. As we continue our research, further refinements and validations on diverse datasets are expected to enhance the model’s performance and generalization capabilities.

**Figure 10:**
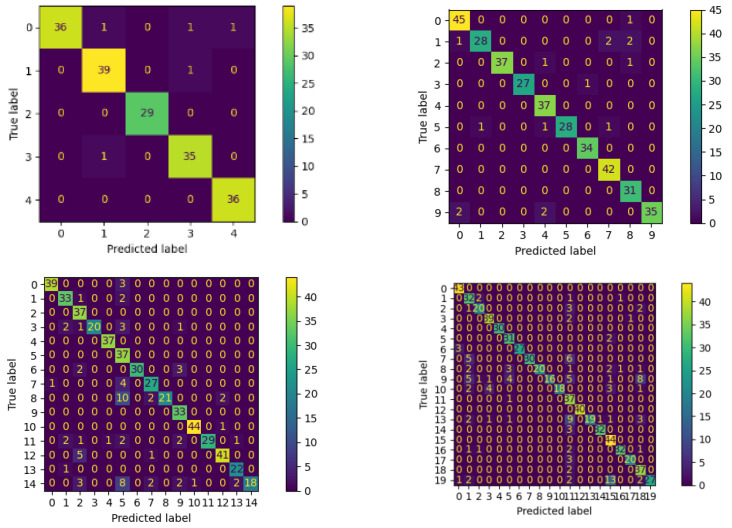
Confusion matrix showing the classification for 5, 10, 15 and 20 subjects.

For a more detailed analysis of the model’s performance, we present the confusion matrix for each case of 5, 10, 15, and 20 subjects, respectively, as shown in the Figure 10. The confusion matrix provides a comprehensive visualization of the model’s classification results, illustrating the distribution of true and predicted labels for each individual. In the confusion matrix, the rows represent the true labels (actual identities of individuals), while the columns represent the predicted labels (model’s identification results). Each entry in the matrix shows the count of instances where a true label corresponds to a specific predicted label. By analyzing the confusion matrices and corresponding graphs, we gain valuable insights into the model’s ability to correctly identify individuals and identify potential misclassifications. The visual representation allows us to observe patterns of correct and incorrect predictions, understand any biases or imbalances in the dataset, and assess the model’s overall performance for personal identification across different subject cases.

**Figure 11:**
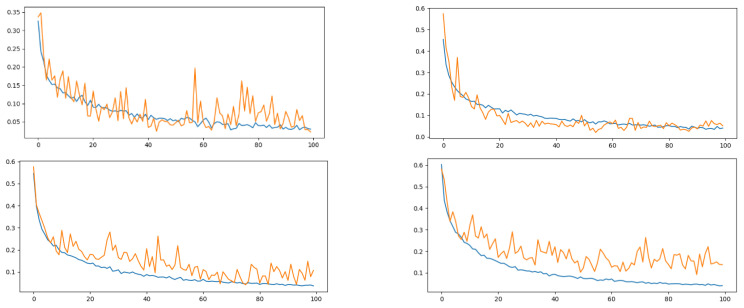
The training (in blue color) and testing (in orange color) accuracies shown of 5, 10, 15, and 20 different subjects with epochs and loss curve (*x*-axis: epochs, *y*-axis: loss curve).

Our ResNet1D-based approach for personal identification using sEMG signals demonstrated remarkable performance across different subject cases. For the case involving five subjects, the training accuracy steadily increased with epochs, indicating the model’s ability to learn from the data and make accurate predictions. Concurrently, the training loss decreased, demonstrating an effective fitting to the training data. Similar trends were observed for testing accuracy and loss, highlighting the model’s capacity to generalize well to unseen data from these five subjects. As we expanded the subject cases to 10, 15, and 20 individuals, the model continued to show commendable progress in both training and testing accuracy, with decreasing loss as depicted in Figure 11. These results underscore the model’s adaptability and generalization capabilities, even in scenarios with 20 subjects, positioning it as a promising solution for real-world applications in healthcare, security, and assistive technologies.

Generally, the accuracy drops when the number of classes increases in a classification problem with any ML model. Though deep neural models, like statistical methods, follow this trend, they can handle the increase in classes when the increase is not significant. In the proposed method, we observed that there was a significant drop in the accuracy as the sEMG signal alone could not differentiate between the subjects as the number of subjects increased. However, when fusing sEMG with other sensor data, the proposed work may perform better, especially in an EHR setting. This will be addressed in future work.

### 4.2. Use-Case Study

An EHR captures, stores, and shares patient data to provide quality healthcare [50]. In response to the escalating volume of patient data, modern technological advancements have facilitated the migration of patient records to cloud databases for streamlined access. A health information system contains sensitive patient information like demographic, specific diagnosis, allergies, and procedures. The massive importance of this sensitive information makes EHRs an attractive target for attacker and hackers. Due to this reason, EHRs pose new challenges and threats to patient privacy and security.

However, this risk can be mitigated by developing a suitable model with standard security and privacy regulations when storing patient data in semi-trusted servers, which ensure patient-centric privacy control over their own EHR. However, electronic health record (EHR) management remains pivotal, demanding secure and efficient approaches. Accessing a patient’s health-related data in a timely manner is critical for prompt and effective treatment. At the primary level, the system allows healthcare professionals to log in with secure and valid authentication to enter patient data into the system. Conventional methods of identification include knowledge-based identifiers and ownership-based ID cards. However, conventional methods entail risks and inconveniences [51]. To eliminate these issues, there is a growing need for biometrics in modern society [52].

Biometrics is a technology that recognizes a user’s information by utilizing unique physical features of their body [53]. Biometric authentication relies on two types of features: physical and behavioral [54]. Physical features include the face, fingerprint, and iris, while behavioral ones encompass the signature, gait, ECG, EMG, and EEG data [55]. Unlike physical features, which can be falsified or altered, behavioral features offer a more secure option. They find applications in personal identification, motion recognition, and disease diagnosis, addressing the limitations of traditional methods.

**Figure 12:**
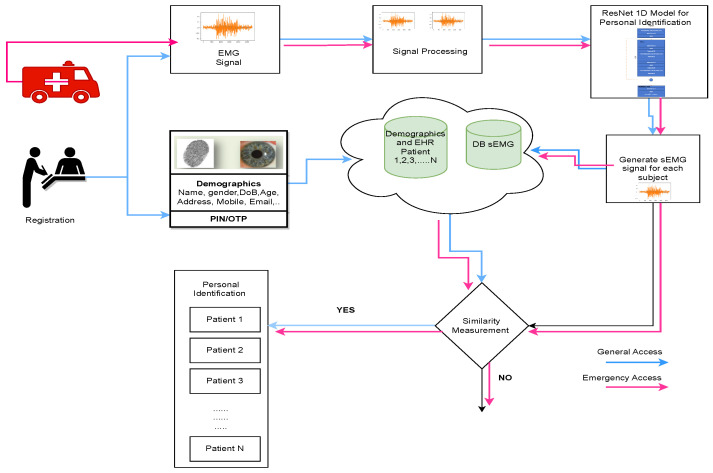
The proposed model for sEMG authentication using general access and emergency access model.

Figure 12 illustrates the proposed model for personal identification using surface electromyography (sEMG) for both general and emergency access to electronic health record (EHR) data.

During patient registration or hospital visits, knowledge-based identifiers and ownership-based ID cards serve as universal patient identifiers. Healthcare user authentication occurs at the database level through valid usernames and passwords. If a user who is not logged in attempts to access a page that requires authentication, they are redirected to a login page where they are prompted to provide their credentials, typically a username and password. In this model, two approaches are employed: the general access model and the emergency access model [56]. In the general access model, both demographic and biometric behavioral features are used for personal identification. To enhance system security, an additional two-step verification process is implemented using biometric behavioral data, specifically the sEMG signal based on the ResNet 1D model. The sEMG signal is generated and stored in the database. During authentication, both levels of verification are performed before granting access to EHR data. In contrast, the emergency access model relies solely on biometric behavioral data from sEMG for patient identification. To enhance patient data security, this innovative application leverages surface (sEMG) signal data stored in the database to determine patient access authorization.

**Figure 13:**
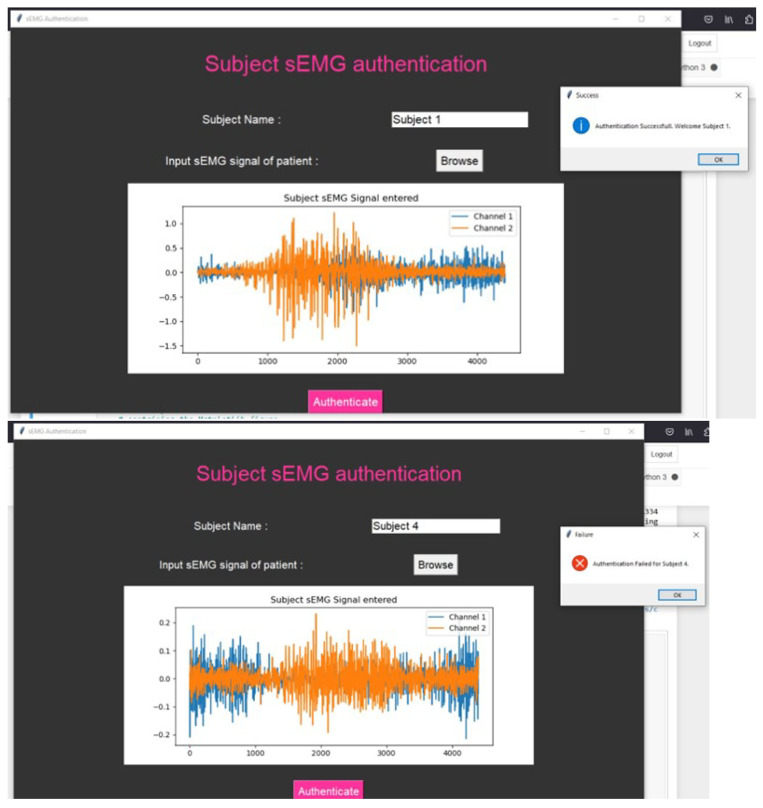
Web-based applications to verify success and failure of authentication.

The web application was specifically designed for the sEMG authentication process as depicted in the Figure 13. During the authentication process, the system verifies whether the sEMG signal corresponds to the same or a different subject. If the sEMG signal does not match the profile of the designated subject, the authentication process fails.

This web application leverages the unique characteristics of sEMG signals for user identification. Each individual’s sEMG signature is distinct due to variations in muscle activity and other physiological factors. By analyzing the sEMG data collected during the authentication attempt, the system can determine if it aligns with the stored reference data for the intended subject. This level of specificity ensures a robust and accurate authentication process. It significantly enhances security, preventing unauthorized access even if an intruder attempts to use someone else’s sEMG signal for authentication. Additionally, the system’s ability to differentiate between sEMG signals from different subjects enhances its accuracy and reliability in confirming the user’s identity. The web application was developed using Python and the Tkinter library; the application operates by invoking a trained model in the background to authenticate entered sEMG signals. This ingenious fusion of biometric identification through sEMG signals bolsters the security of patient data access, exemplifying the integration of cutting-edge technology to ensure the utmost privacy and safeguarding of sensitive medical records.

## 5. Conclusions

This research highlighted the significant potential of ResNet-1D neural networks in the analysis of sEMG signals, particularly for personal identification. The dataset utilized in this study, known as CU_sEMG DB, was developed by the IT Research Institute of Chosun University and consisted of data from 200 subjects performing 12 distinct gestures. Each gesture was repeated ten times across three sessions with intervals of at least a day. In our study, we applied ResNet-1D architectures and conducted extensive performance analysis involving 5, 10, 15, and 20 subjects. By harnessing the deep feature learning capabilities of ResNet-1D and its proficiency in capturing intricate temporal patterns, we demonstrated its effectiveness in personal identification tasks.

The use of biological signals for user authentication is poised for widespread adoption due to the inherent security they offer. We developed a web-based sEMG application to evaluate the system’s ability to distinguish between sEMG signals from different subjects, thereby enhancing its accuracy and reliability in confirming users’ identities. The findings presented in this study not only underscore the promise of deep learning techniques in sEMG signal analysis but also pave the way for future advancements in this field. This progress holds substantial potential for enhancing the security and precision of sEMG-based applications, contributing to their continued development and adoption. 

## Figures and Tables

**Table 1:** Tabulation value of model performance metrics of 5 and 10 subjects.

Subject IDs	Recall	Precision	F1-Score
0	0.92	1.00	0.96
1	0.97	0.95	0.96
2	1.00	1.00	1.00
3	0.97	0.95	0.96
4	1.00	0.97	0.99
Accuracy	-	-	0.97
Macro avg	0.97	0.97	0.97
Weighted avg	0.97	0.97	0.97
0	0.98	0.94	0.96
1	1.00	0.93	0.97
2	1.00	0.97	0.99
3	0.90	1.00	0.95
4	0.96	1.00	0.98
5	0.90	1.00	0.95
6	0.85	0.97	0.90
7	1.00	0.90	0.95
8	0.95	1.00	0.97
9	1.00	0.89	0.94
Accuracy	-	-	0.96
Macro avg	0.95	0.96	0.96
Weighted avg	0.96	0.96	0.96

**Table 2:** Tabulation value of model performance metrics of 15 and 20 subjects.

Subject IDs	Recall	Precision	F1-Score
0	0.92	0.87	0.89
1	0.98	0.98	0.98
2	1.00	0.80	0.89
3	0.50	1.00	0.67
4	0.86	1.00	0.92
5	0.60	1.00	0.75
6	0.74	1.00	0.85
7	0.84	0.84	0.84
8	1.00	0.97	0.99
9	0.76	1.00	0.87
10	1.00	0.54	0.70
11	1.00	0.74	0.85
12	0.93	0.97	0.95
13	0.96	0.88	0.92
14	0.87	0.93	0.90
Accuracy	-	-	0.87
Macro avg	0.86	0.90	0.86
Weighted avg	0.90	0.87	0.87
0	0.89	0.62	0.73
1	1.00	1.00	1.00
2	0.97	0.49	0.65
3	0.57	1.00	0.73
4	0.73	1.00	0.85
5	0.64	1.00	0.78
6	0.93	0.87	0.90
7	0.67	1.00	1.00
8	1.00	1.00	1.00
9	0.51	1.00	0.68
10	0.94	0.78	0.85
11	0.77	0.83	0.80
12	1.00	0.91	0.96
13	0.87	0.95	0.91
14	0.86	0.94	0.90
15	0.95	0.67	0.79
16	0.90	1.00	0.95
17	0.91	0.97	0.94
18	1.00	0.67	0.80
19	0.39	1.00	0.56
Accuracy	-	-	0.82
Macro avg	0.83	0.88	0.83
Weighted avg	0.88	0.82	0.83

## Data Availability

The data supporting the findings presented in this article are available at https://www.chosun.ac.kr/user/indexSub.do?codyMenuSeq=940232546&siteId=riit (accessed on 21 February 2023).

## Abbreviations

The following abbreviations are used in this manuscript:

sEMG	Surface electromyography
DB	Database
DNA	Deoxyribonucleic Acid
EMG	Electromyography
GRU	Gated Recurrent Unit
NF	Notch filter
LSTM	Long Short-Term Memory
CNN	Convolutional Neural Network
